# 3-Allyl-2-hydr­oxy-5,6,8-trimethoxy­naphthalene-1,4-dione

**DOI:** 10.1107/S1600536808028432

**Published:** 2008-09-13

**Authors:** Dominea C. K. Rathwell, Kit Y. Tsang, Ka Wai Choi, Peter D. W. Boyd, Margaret A. Brimble

**Affiliations:** a Department of Chemistry, University of Auckland, Private Bag 92019, Auckland, New Zealand

## Abstract

In the crystal structure of the title compound, C_16_H_16_O_6_, a pair of naphthoquinone rings are linked *via* O—H⋯O—C hydrogen bonds in a nearly orthogonal arrangement. This dimeric unit is linked to a neighbouring dimer by π–π stacking inter­actions between the naphthoquinone rings, where the distance between the mean plane of the naphtoquinone backbones is 3.468 Å, and O—H⋯O—C hydrogen bonds.

## Related literature

For details of the synthesis, see: Brimble *et al.* (2008 ▶). For related syntheses, see: Reissig *et al.* (2006 ▶); Kozlowski *et al.* (2008 ▶). For the biological activity of rubromycins, see: Brockmann *et al.* (1953 ▶, 1966 ▶).
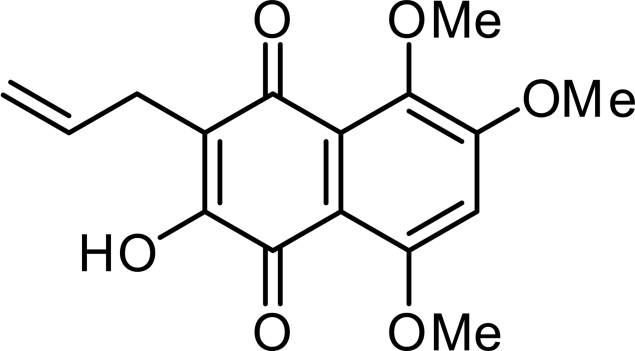


## Experimental

### 

#### Crystal data

C_16_H_16_O_6_
*M* *_r_* = 304.29
Orthorhombic, 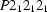

*a* = 4.68110 (10) Å
*b* = 12.6577 (3) Å
*c* = 23.3392 (5) Å
*V* = 1382.89 (5) Å^3^
*Z* = 4
Mo *K*α radiation
μ = 0.11 mm^−1^
*T* = 89 (2) K
0.28 × 0.09 × 0.06 mm

#### Data collection

Bruker SMART diffractometer with APEXII CCD detector
Absorption correction: none
14372 measured reflections
1914 independent reflections
1415 reflections with *I* > 2σ(*I*)
*R*_int_ = 0.031

#### Refinement

*R*[*F* ^2^ > 2σ(*F* ^2^)] = 0.040
*wR*(*F* ^2^) = 0.085
*S* = 1.09
1914 reflections
200 parameters
H-atom parameters constrained
Δρ_max_ = 0.22 e Å^−3^
Δρ_min_ = −0.28 e Å^−3^

### 

Data collection: *APEX2* (Bruker, 2005 ▶); cell refinement: *APEX2*; data reduction: *SAINT* (Bruker, 2005 ▶); program(s) used to solve structure: *SHELXS97* (Sheldrick, 2008 ▶); program(s) used to refine structure: *SHELXL97* (Sheldrick, 2008 ▶); molecular graphics: *ORTEPIII* (Burnett & Johnson, 1996 ▶) and *Mercury* (Macrae *et al.*, 2006 ▶); software used to prepare material for publication: *WinGX* (Farrugia, 1999 ▶) and *publCIF* (Westrip, 2008 ▶).

## Supplementary Material

Crystal structure: contains datablocks global, I. DOI: 10.1107/S1600536808028432/ng2489sup1.cif

Structure factors: contains datablocks I. DOI: 10.1107/S1600536808028432/ng2489Isup2.hkl

Additional supplementary materials:  crystallographic information; 3D view; checkCIF report

## Figures and Tables

**Table 1:** Hydrogen-bond geometry (Å, °)

*D*—H⋯*A*	*D*—H	H⋯*A*	*D*⋯*A*	*D*—H⋯*A*
O16—H16⋯O12	0.82	2.14	2.612 (2)	117
O16—H16⋯O12^i^	0.82	2.05	2.777 (2)	148
O16—H16⋯O19^i^	0.82	2.40	2.926 (2)	122

Symmetry code: (i) 
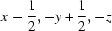
.

**Fig. 1.**
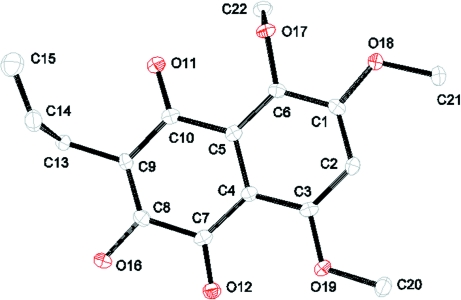
The molecular structure and the atom numbering scheme of the title compound. Ellipsoids are drawn at 50% probability level for non-H atoms.

**Fig. 2.**
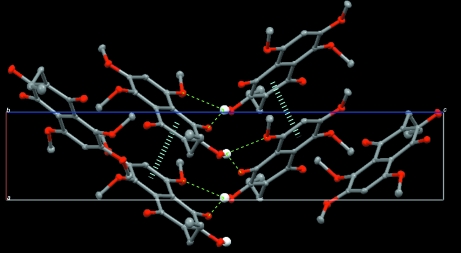
Molecular packing of the title naphthoquinone, viewed along the b axis. (···, in green) hydrogen bond; (|||, in blue) π–π interaction. The H atoms not involved in hydrogen bonding have been omitted.

## supplementary crystallographic information

### Comment

The rubromycins are a structurally related family of antibiotics that exhibit a wide range of biological activity (Brockmann *et al.*, 1953, 1966). The common structural features of this family of antibiotics consists of a naphthoquinone and an isocoumarin ring linked through a bis-benzannelated-5,6-spiroacetal ring system. Our recent synthetic efforts have focused on the synthesis of the naphthoquinone units of the rubromycins and its regioisomeric counterpart. The tandem Ullman coupling–Claisen rearrangement has been successfully employed to access the regioisomeric naphthoquinones in which the title compound was isolated as a minor isomer. The newly introduced hydroxyl and allyl groups were established by X-ray crystallography to be at C8 and C9, respectively (Fig. 1). The crystal packing is dominated by intermolecular hydrogen bonds and π–π interactions (Table 1, Fig. 2). Further detailed analysis revealed that a pair of naphthoquinone rings are linked *via* O—H···O—C hydrogen bonding in a near orthogonal arrangement with respect to each other. This dimeric unit stacks on top of a neighbouring dimer with π–π stacking interactions between the naphthoquinone rings and O—H···O—C hydrogen bonding (Table 1, Fig. 2).

### Experimental

A mixture of 2-bromo-5,7,8-trimethoxynaphthalene-1,4-dione (500 mg, 1.6 mmol), allyl alcohol (0.5 ml, 10 mmol), copper iodide (46 mg, 0.14 mmol) and caesium carbonate (940 mg, 2.9 mmol) in toluene (3 ml) was heated at 320 K under a nitrogen atmosphere in a sealed tube for 30 min. After allowing the mixture to cool to room temperature, the brownish mixture was filtered through a plug of Celite. The brown filtrate was then irradiated with microwave at 410 K (60 W) for 180 min in a sealed tube (10 ml pressure-rated reaction vial) in a self-tuning single mode irradiating synthesizer (CEM Discover LabMate microwave synthesizer). The resulting solution was then concentrated *in vacuo* to afford a brown residue. Purification of the crude residue by flash column chromatography using ethyl acetate–hexane (2:8) with gradient elution to neat ethyl acetate afforded the title compound (116 mg, 24%) as a yellow solid. Recrystallization from acetonitrile afforded yellow needles suitable for X-ray diffraction. m.p. 427–431 K

### Refinement

Hydrogen atoms were placed in calculated positions and refined using the riding model (C—H 0.93–0.97 Å), with *U*_iso_(H) = 1.5 times *U*_eq_(O) and *U*_iso_(H) = 1.2 or 1.5 times *U*_eq_(C).

### Crystal data

**Table:**

C_16_H_16_O_6_	*D*_x_ = 1.462 Mg m^−^^3^
*M**_r_* = 304.29	Melting point: 429(2) K
Orthorhombic, *P*2_1_2_1_2_1_	Mo *K*α radiation, λ = 0.71073 Å
*a* = 4.6811 (1) Å	Cell parameters from 3251 reflections
*b* = 12.6577 (3) Å	θ = 1.8–27.9°
*c* = 23.3392 (5) Å	µ = 0.11 mm^−^^1^
*V* = 1382.89 (5) Å^3^	*T* = 89 K
*Z* = 4	Needle, yellow
*F*(000) = 640	0.28 × 0.09 × 0.06 mm

### Data collection

**Table:**

Bruker SMART diffractometer with APEXII CCD detector	1415 reflections with *I* > 2σ(*I*)
Radiation source: fine-focus sealed tube	*R*_int_ = 0.031
graphite	θ_max_ = 27.9°, θ_min_ = 1.8°
ω scans	*h* = −6→4
14372 measured reflections	*k* = −16→16
1914 independent reflections	*l* = −30→30

### Refinement

**Table:**

Refinement on *F*^2^	Primary atom site location: structure-invariant direct methods
Least-squares matrix: full	Secondary atom site location: difference Fourier map
*R*[*F*^2^ > 2σ(*F*^2^)] = 0.040	Hydrogen site location: inferred from neighbouring sites
*wR*(*F*^2^) = 0.085	H-atom parameters constrained
*S* = 1.09	*w* = 1/[σ^2^(*F*_o_^2^) + (0.0367*P*)^2^ + 0.1796*P*] where *P* = (*F*_o_^2^ + 2*F*_c_^2^)/3
1914 reflections	(Δ/σ)_max_ < 0.001
200 parameters	Δρ_max_ = 0.22 e Å^−^^3^
0 restraints	Δρ_min_ = −0.28 e Å^−^^3^

### Special details

**Table:**

Geometry. All e.s.d.'s (except the e.s.d. in the dihedral angle between two l.s. planes) are estimated using the full covariance matrix. The cell e.s.d.'s are taken into account individually in the estimation of e.s.d.'s in distances, angles and torsion angles; correlations between e.s.d.'s in cell parameters are only used when they are defined by crystal symmetry. An approximate (isotropic) treatment of cell e.s.d.'s is used for estimating e.s.d.'s involving l.s. planes.
Refinement. Refinement of *F*^2^ against ALL reflections. The weighted *R*-factor *wR* and goodness of fit *S* are based on *F*^2^, conventional *R*-factors *R* are based on *F*, with *F* set to zero for negative *F*^2^. The threshold expression of *F*^2^ > σ(*F*^2^) is used only for calculating *R*-factors(gt) *etc*. and is not relevant to the choice of reflections for refinement. *R*-factors based on *F*^2^ are statistically about twice as large as those based on *F*, and *R*- factors based on ALL data will be even larger.

### Fractional atomic coordinates and isotropic or equivalent isotropic displacement parameters (Å^2^)

**Table:**

	*x*	*y*	*z*	*U*_iso_*/*U*_eq_	
O16	−0.4858 (4)	0.07396 (13)	0.01234 (6)	0.0192 (4)	
H16	−0.4751	0.1330	−0.0020	0.029*	
O11	−0.1507 (4)	−0.09214 (12)	0.17755 (7)	0.0183 (4)	
O17	0.2562 (4)	−0.00627 (12)	0.24216 (6)	0.0150 (4)	
O19	0.2115 (4)	0.33797 (12)	0.09662 (7)	0.0174 (4)	
O12	−0.1853 (4)	0.24133 (13)	0.03789 (6)	0.0164 (4)	
O18	0.5482 (4)	0.16101 (12)	0.26649 (6)	0.0162 (4)	
C5	0.0492 (6)	0.07747 (18)	0.15754 (9)	0.0125 (6)	
C4	0.0441 (6)	0.16715 (18)	0.12052 (9)	0.0129 (5)	
C2	0.3969 (6)	0.25329 (19)	0.18127 (10)	0.0141 (6)	
H2	0.5140	0.3108	0.1891	0.017*	
C6	0.2263 (6)	0.07808 (18)	0.20552 (9)	0.0129 (6)	
C3	0.2219 (6)	0.25382 (18)	0.13284 (9)	0.0134 (6)	
C8	−0.3187 (6)	0.06946 (19)	0.05919 (9)	0.0148 (6)	
C21	0.7270 (6)	0.24990 (19)	0.28100 (10)	0.0180 (6)	
H21A	0.8237	0.2360	0.3165	0.027*	
H21B	0.6110	0.3120	0.2850	0.027*	
H21C	0.8653	0.2608	0.2512	0.027*	
C9	−0.3109 (6)	−0.01717 (18)	0.09232 (9)	0.0135 (6)	
C1	0.3956 (6)	0.16712 (19)	0.21750 (9)	0.0130 (6)	
C7	−0.1488 (6)	0.16625 (19)	0.07100 (9)	0.0141 (6)	
C20	0.4222 (6)	0.41995 (19)	0.10271 (10)	0.0196 (6)	
H20A	0.3889	0.4738	0.0745	0.029*	
H20B	0.6093	0.3906	0.0973	0.029*	
H20C	0.4089	0.4502	0.1403	0.029*	
C10	−0.1373 (6)	−0.01652 (19)	0.14485 (9)	0.0134 (6)	
C14	−0.2956 (7)	−0.2026 (2)	0.05618 (11)	0.0214 (7)	
H14	−0.2007	−0.1904	0.0218	0.026*	
C22	0.0511 (6)	−0.00647 (18)	0.28793 (9)	0.0175 (6)	
H22A	0.0825	−0.0669	0.3120	0.026*	
H22B	−0.1383	−0.0095	0.2722	0.026*	
H22C	0.0719	0.0568	0.3102	0.026*	
C15	−0.2583 (7)	−0.2953 (2)	0.08090 (11)	0.0288 (8)	
H15A	−0.3497	−0.3104	0.1153	0.035*	
H15B	−0.1405	−0.3455	0.0639	0.035*	
C13	−0.4799 (6)	−0.11548 (17)	0.07933 (10)	0.0155 (6)	
H13A	−0.5731	−0.1396	0.1141	0.019*	
H13B	−0.6273	−0.0988	0.0516	0.019*	

### Atomic displacement parameters (Å^2^)

**Table:**

	*U*^11^	*U*^22^	*U*^33^	*U*^12^	*U*^13^	*U*^23^
O16	0.0252 (12)	0.0179 (9)	0.0146 (8)	−0.0020 (10)	−0.0054 (9)	0.0036 (7)
O11	0.0207 (11)	0.0155 (9)	0.0185 (8)	−0.0029 (9)	−0.0019 (8)	0.0038 (7)
O17	0.0168 (10)	0.0133 (8)	0.0148 (8)	0.0008 (9)	0.0016 (9)	0.0044 (7)
O19	0.0200 (11)	0.0132 (8)	0.0190 (9)	−0.0049 (9)	−0.0026 (9)	0.0042 (7)
O12	0.0164 (10)	0.0170 (9)	0.0157 (8)	−0.0003 (9)	0.0003 (9)	0.0024 (7)
O18	0.0178 (10)	0.0136 (8)	0.0171 (8)	−0.0029 (9)	−0.0039 (9)	0.0014 (7)
C5	0.0116 (15)	0.0122 (11)	0.0136 (11)	0.0032 (12)	0.0041 (12)	−0.0012 (9)
C4	0.0125 (14)	0.0135 (11)	0.0127 (11)	−0.0004 (12)	0.0021 (12)	0.0010 (9)
C2	0.0120 (15)	0.0130 (12)	0.0172 (12)	−0.0016 (12)	0.0018 (12)	−0.0018 (10)
C6	0.0130 (15)	0.0137 (12)	0.0121 (11)	0.0032 (13)	0.0011 (12)	0.0010 (9)
C3	0.0133 (15)	0.0118 (12)	0.0149 (11)	0.0030 (13)	0.0032 (13)	0.0023 (9)
C8	0.0130 (15)	0.0191 (13)	0.0122 (11)	0.0008 (13)	0.0000 (12)	−0.0005 (10)
C21	0.0190 (17)	0.0171 (13)	0.0179 (12)	−0.0043 (14)	−0.0045 (14)	−0.0024 (10)
C9	0.0118 (15)	0.0152 (12)	0.0136 (11)	−0.0009 (12)	−0.0013 (12)	−0.0013 (10)
C1	0.0102 (15)	0.0166 (12)	0.0120 (11)	0.0051 (12)	0.0004 (11)	−0.0014 (10)
C7	0.0135 (15)	0.0149 (12)	0.0140 (11)	0.0009 (13)	0.0056 (12)	0.0011 (10)
C20	0.0220 (17)	0.0168 (13)	0.0198 (12)	−0.0052 (13)	0.0005 (13)	−0.0011 (11)
C10	0.0118 (15)	0.0139 (12)	0.0144 (11)	0.0033 (11)	0.0039 (12)	0.0001 (10)
C14	0.0233 (18)	0.0216 (14)	0.0193 (12)	−0.0058 (14)	−0.0010 (14)	−0.0031 (10)
C22	0.0211 (15)	0.0171 (13)	0.0142 (11)	−0.0003 (13)	0.0024 (13)	0.0034 (10)
C15	0.036 (2)	0.0226 (15)	0.0275 (14)	0.0036 (16)	0.0000 (17)	−0.0033 (12)
C13	0.0155 (16)	0.0153 (12)	0.0156 (11)	−0.0041 (12)	−0.0031 (14)	0.0027 (10)

### Geometric parameters (Å, °)

**Table:**

O16—C8	1.346 (3)	C8—C7	1.486 (3)
O16—H16	0.8200	C21—H21A	0.9600
O11—C10	1.226 (3)	C21—H21B	0.9600
O17—C6	1.375 (3)	C21—H21C	0.9600
O17—C22	1.436 (3)	C9—C10	1.471 (3)
O19—C3	1.361 (3)	C9—C13	1.505 (3)
O19—C20	1.439 (3)	C20—H20A	0.9600
O12—C7	1.237 (3)	C20—H20B	0.9600
O18—C1	1.350 (3)	C20—H20C	0.9600
O18—C21	1.443 (3)	C14—C15	1.318 (4)
C5—C6	1.394 (3)	C14—C13	1.501 (4)
C5—C4	1.427 (3)	C14—H14	0.9300
C5—C10	1.505 (3)	C22—H22A	0.9600
C4—C3	1.407 (3)	C22—H22B	0.9600
C4—C7	1.467 (3)	C22—H22C	0.9600
C2—C1	1.380 (3)	C15—H15A	0.9300
C2—C3	1.396 (3)	C15—H15B	0.9300
C2—H2	0.9300	C13—H13A	0.9700
C6—C1	1.406 (3)	C13—H13B	0.9700
C8—C9	1.342 (3)		
			
C8—O16—H16	109.5	O18—C1—C6	114.9 (2)
C6—O17—C22	113.32 (18)	C2—C1—C6	120.9 (2)
C3—O19—C20	118.60 (19)	O12—C7—C4	124.8 (2)
C1—O18—C21	117.46 (18)	O12—C7—C8	116.3 (2)
C6—C5—C4	119.5 (2)	C4—C7—C8	118.8 (2)
C6—C5—C10	120.5 (2)	O19—C20—H20A	109.5
C4—C5—C10	120.0 (2)	O19—C20—H20B	109.5
C3—C4—C5	119.1 (2)	H20A—C20—H20B	109.5
C3—C4—C7	122.1 (2)	O19—C20—H20C	109.5
C5—C4—C7	118.8 (2)	H20A—C20—H20C	109.5
C1—C2—C3	119.8 (2)	H20B—C20—H20C	109.5
C1—C2—H2	120.1	O11—C10—C9	119.1 (2)
C3—C2—H2	120.1	O11—C10—C5	121.6 (2)
O17—C6—C5	123.8 (2)	C9—C10—C5	119.3 (2)
O17—C6—C1	116.2 (2)	C15—C14—C13	124.9 (3)
C5—C6—C1	120.0 (2)	C15—C14—H14	117.5
O19—C3—C2	121.9 (2)	C13—C14—H14	117.5
O19—C3—C4	117.5 (2)	O17—C22—H22A	109.5
C2—C3—C4	120.6 (2)	O17—C22—H22B	109.5
C9—C8—O16	121.2 (2)	H22A—C22—H22B	109.5
C9—C8—C7	123.5 (2)	O17—C22—H22C	109.5
O16—C8—C7	115.26 (19)	H22A—C22—H22C	109.5
O18—C21—H21A	109.5	H22B—C22—H22C	109.5
O18—C21—H21B	109.5	C14—C15—H15A	120.0
H21A—C21—H21B	109.5	C14—C15—H15B	120.0
O18—C21—H21C	109.5	H15A—C15—H15B	120.0
H21A—C21—H21C	109.5	C14—C13—C9	112.2 (2)
H21B—C21—H21C	109.5	C14—C13—H13A	109.2
C8—C9—C10	119.4 (2)	C9—C13—H13A	109.2
C8—C9—C13	123.0 (2)	C14—C13—H13B	109.2
C10—C9—C13	117.6 (2)	C9—C13—H13B	109.2
O18—C1—C2	124.2 (2)	H13A—C13—H13B	107.9

### Hydrogen-bond geometry (Å, °)

**Table:**

*D*—H···*A*	*D*—H	H···*A*	*D*···*A*	*D*—H···*A*
O16—H16···O12	0.82	2.14	2.612 (2)	117.
O16—H16···O12^i^	0.82	2.05	2.777 (2)	148.
O16—H16···O19^i^	0.82	2.40	2.926 (2)	122.

Symmetry codes: (i) *x*−1/2, −*y*+1/2, −*z*.
